# Supplementary material for: Development of a Combined Lipid-Based Nanoparticle Formulation for Enhanced siRNA Delivery to Vascular Endothelial Cells
Source: Pharmaceutics. 2022 Sep 29;14(10):2086. doi: 10.3390/pharmaceutics14102086 (PMC9609090; doi:10.3390/pharmaceutics14102086)
Supplement: Supplementary file 1 [file pharmaceutics-14-02086-s001.zip › pharmaceutics-1931891-supplementary.pdf]

Supplementary Materials

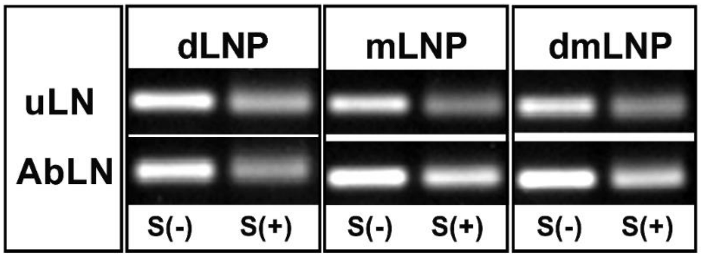

Figure S1. Agarose gel electrophoresis image of siRNA encapsulated in uncoupled or Ab_{VCAM-1} coupled LNPs (uLN or AbLN), which were stored for 3 weeks at 4°C. The three LNP formulations that were tested, were dLNP: the LNP formulation with 50 mol% DOTAP, mLNP: the LNP formulation with 50 mol% MC3, and dmLNP: the LNP formulation with 10 mol% DOTAP and 40 mol% MC3. The LNP samples were incubated with 40% serum (+) or without serum (-) for 1.5 h at 37°C.

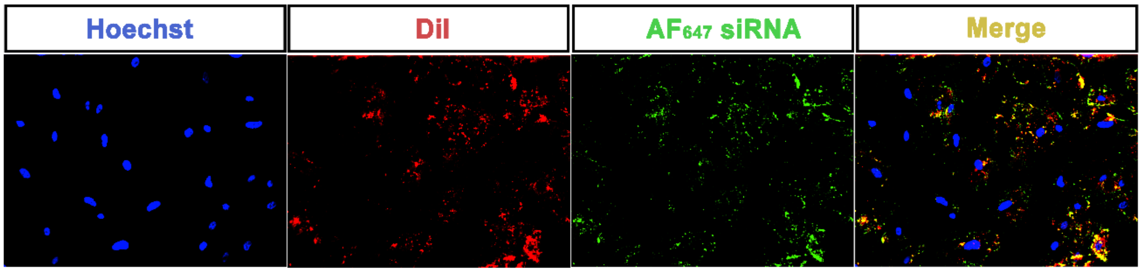

Figure S2. Intracellular release of siRNA from Ab_{VCAM-1} coupled mLNP in endothelial cells. HUVEC were incubated with Ab_{VCAM-1} coupled mLNP labeled with DiI (red) and loaded with $AlexaFluor_{647}$ siRNA (green) for 6 h. The nuclei were stained and visualized as blue.

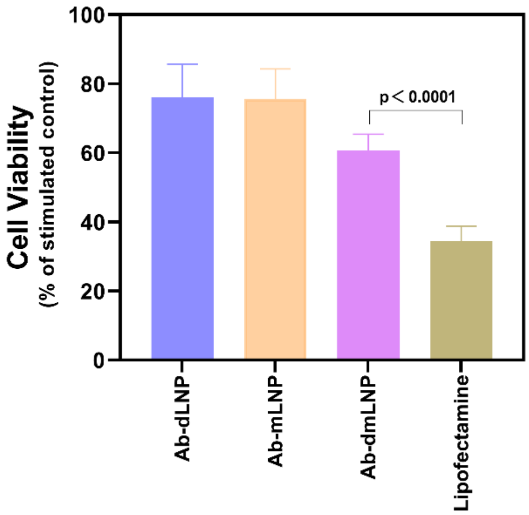

Figure S3. Cell viability of HUVEC transfected with Ab_{VCAM-1} coupled LNP or lipofectamine. TNF-α activated HUVEC were transfected with Ab_{VCAM-1} coupled LNP or lipofectamine containining siRNA for 24 h, at siRNA

concentration of 60nM. Cell viability was measured using CCK-8 assay, as described in the 'Material and Methods'section.

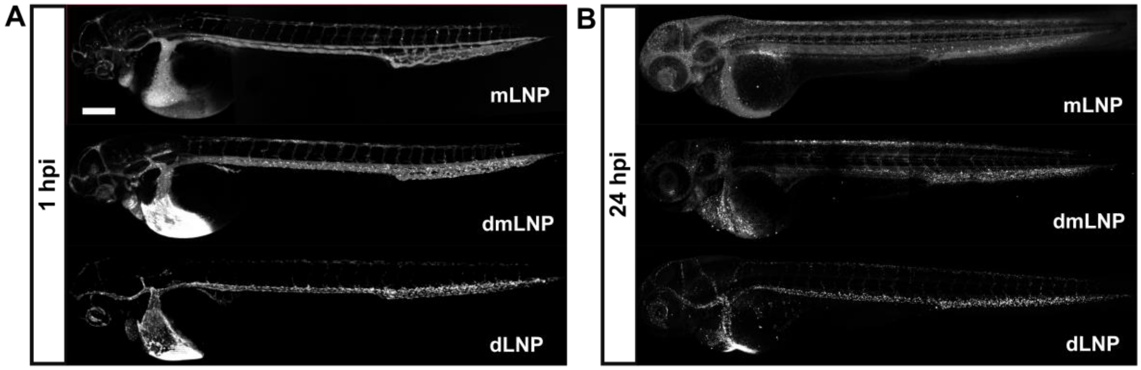

Figure S4. Biodistribution of mLNP, dmLNP, and dLNP at the indicated hour post-injection of (A) 1hpi and (B) 24 hpi in 54 hpf zebrafish embryos at whole-embryo view (scale bar: 200 μm).

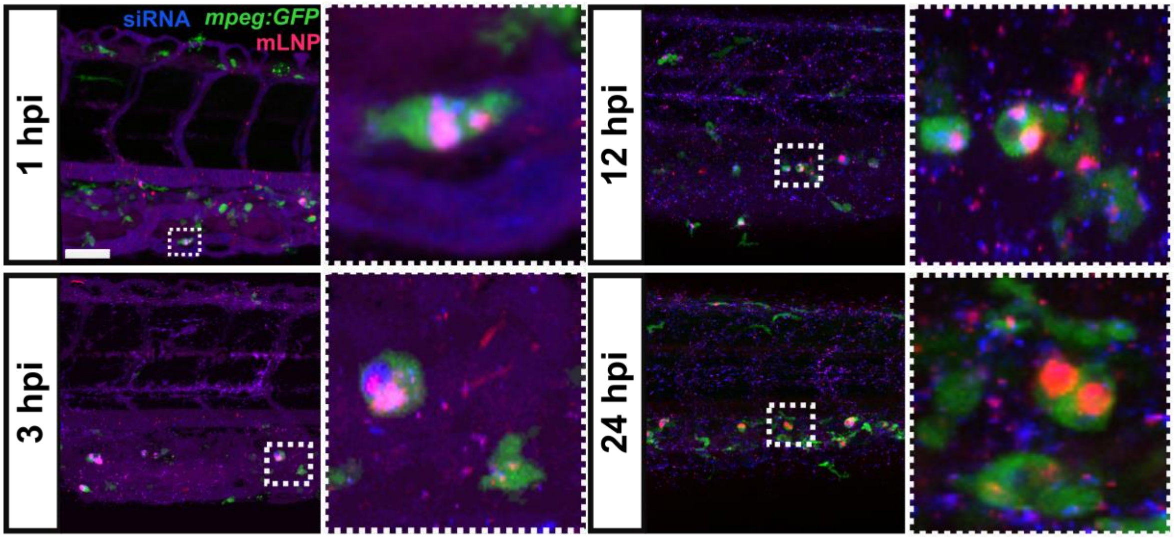

Figure S5. Biodistribution of mLNP (red) and siRNA (blue) in 54 hpf *mpeg*:eGFP zebrafish embryos (macrophages in green). Scale bar: 50 μm. White boxes show the zoom-in areas.

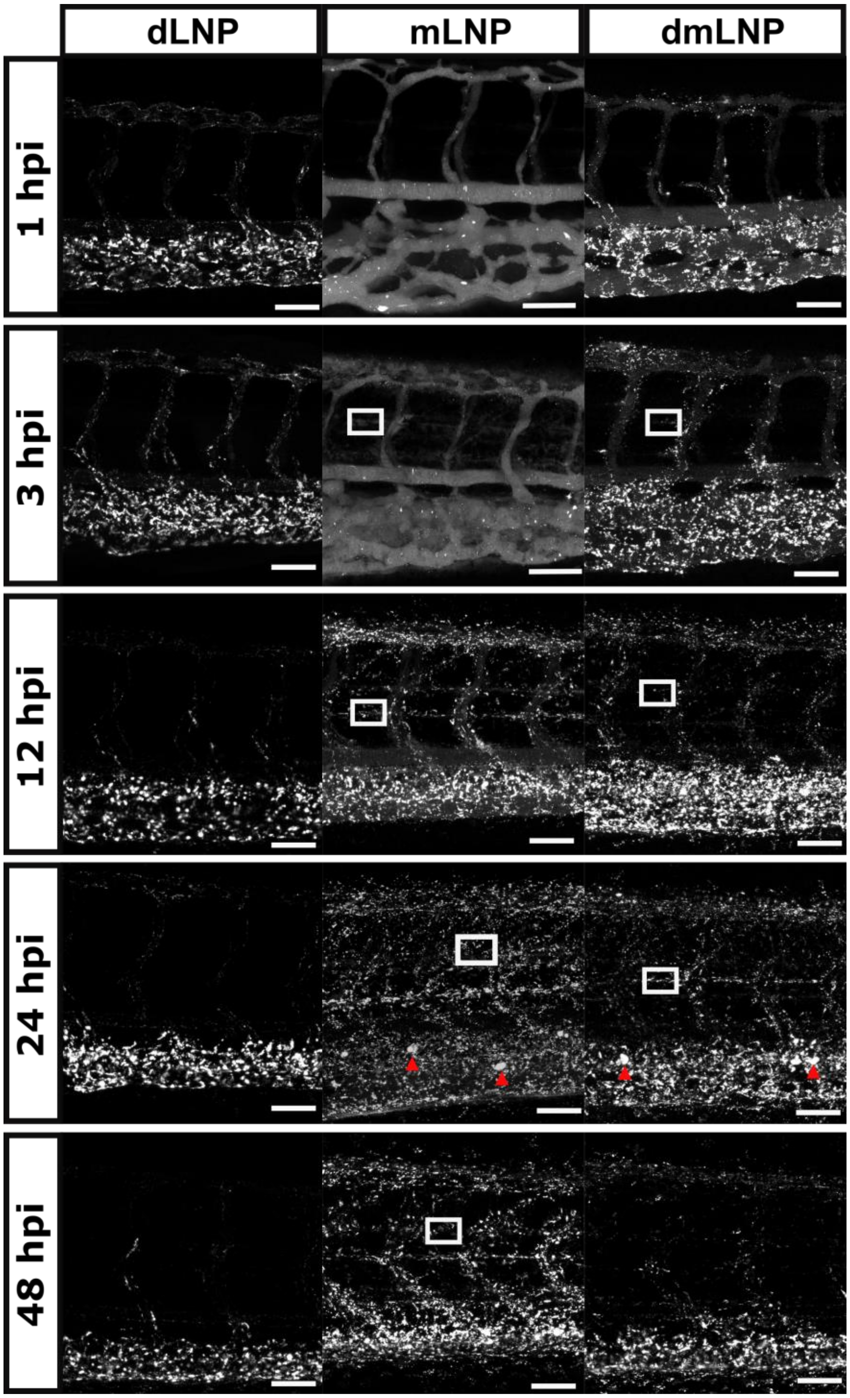

Figure S6. Biodistribution of siRNA (white) associated with different LNP formulations in 54 hpf zebrafish embryos at tissue-level view (scale bar: 50 μm). Macrophage-uptake of LNP is marked with red triangles. Extravasation of LNP is indicated by white rectangles.

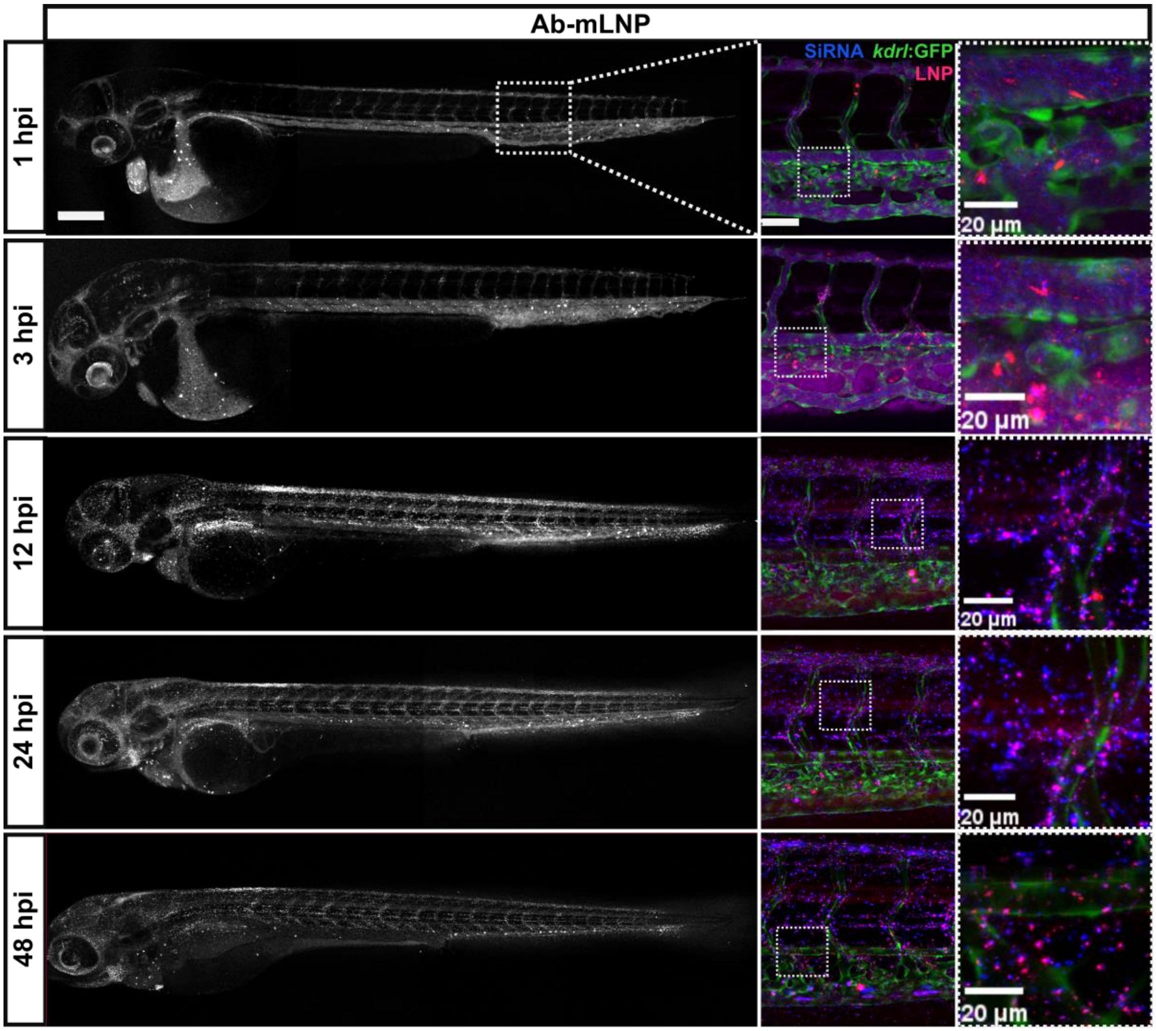

Figure S7. Biodistribution of control antibody-coupled mLNP and associated siRNA in 54 hpf *kdrl*:eGFP zebrafish embryos at various time points. The whole-embryo shows (scale bar: 200 µm) Ab-mLNP distribution (in white), and the tissue-level shows (scale bar: 50 µm) the distribution of Ab-LNP (red) and siRNA (blue) in blood vessels (green).
